# Partial‐least‐squares and canonical‐correlation analysis of chemical constituents and active ingredients of new types of Chinese mulberries

**DOI:** 10.1002/fsn3.753

**Published:** 2018-09-07

**Authors:** Rui Sun, Lei Sun, Chuanming Han

**Affiliations:** ^1^ School of Food Science and Engineering Qilu University of Technology (Shandong Academy of Sciences) Jinan China; ^2^ Economic Forest Institute Shandong Academy of Forestry Sciences Jinan China

**Keywords:** anthocyanin rutin, canonical correlation, chemical composition, mineral element, mulberry, partial‐least‐squares analysis, principle‐component analysis

## Abstract

**Objective:**

To investigate the correlation between chemical constituents and active ingredients of 13 types of Chinese mulberry fruits.

**Methods:**

Thirteen types mulberry fruits were harvested. The correlation between chemical constituents and active ingredients (primarily anthocyanins and rutins) of 13 new types of Chinese mulberries was assessed using partial‐least‐squares, principle‐component and canonical‐correlation analyses.

**Results:**

Vitamin C and titratable acid in the mulberry fruits were critical components that affected the active ingredients, especially anthocyanins and rutins. The content of titratable acid content was related to the fruit flavor and maintained the balance of anthocyanins, vitamin C and rutins. Mineral elements, such as Zn and Cu, also played a vital role in these processes. Low contents of sugar, crude protein, crude fat and pectin were significantly correlated with the mineral elements.

**Conclusion:**

Chemical constituents and mineral elements can mutually affect the concentration. It provides a novel method for any changes in the quality of new types of Chinese mulberries, which can identify the sources of new types of natural antioxidants.

## 1 INTRODUCTION

Berries, including mulberries, are regarded as optimal sources of beneficial chemical compounds. The mulberries, belonging to the *Moris* genus of the *Moraceae*family, are deciduous cone‐shaped trees approximately 10 to 13 m in height (Kostiæ et al., 2013; Lin & Lay, 2013).The mulberries have adapted to grow in a wide range of climatic, topographic, and soil conditions from the temperate to subtropical regions of the northern hemisphere and in the tropics of the southern hemisphere (Özgen, Serçe, & Kaya, 2009; Zerega, Clement, Datwyler, & Weiblen, 2005).

China is home to 15 mulberry species with four varieties (Tian, Zhu, & Lin, 2010; Zhao et al., 2007) consisting of over 3,000 genetic mulberry resources, the largest quantity of mulberry species worldwide. The demand for the most tasty and palatable fruit has been changed along with the extended application scope of mulberries. Through decades of planting, selection and introduction, over 10 new mulberry cultivars with tasty fruits have been selected and unique seed resources for planting have been cultivated in China (Chen, Kan, Tang, Cai, & Liu, 2012).

Mulberry foliage is used to feed silkworms and the mulberry fruit is widely consumed due to the chemical composition with high biological activity (Ercisli & Orhan, 2007; Natić et al., 2015). Mulberries and mulberry leaves are high in anthocyanins and flavonoids, such as rutin (Boranbayeva, Karadeniz, & Yılmaz, 2014; Du, Zheng, & Xu, 2008; Zhang, Han, He, & Duan, 2008), minerals (Ercisli & Orhan, 2007), phenol acids, amino acids, alkaloids (Asano et al., 2001; Hassimotto, Genovese, & Lajolo, 2007), carotenoids (Arabshahi‐Delouee & Urooj, 2007), sugars (glucose and fructose), vitamins, trace elements, and fats (mainly linoleic acid, palmitic acid and oleic acid). All these components render them with a wide range of biopharmaceutical activities including anti‐diabetic, anti‐bacterial, anti‐cancer, cardiovascular, hypolipidemic, anti‐oxidant, anti‐atherogenic, and anti‐inflammatory properties (Gryn‐Rynkoa, Bazylaka, & Olszewska‐Sloninab, 2016; Jiang et al., 2013). Mature mulberry contains a high concentration of water‐soluble pigments, anthocyanins, primarily cyanidin‐3‐glucoside and cyanidin‐3‐rutinoside (Suh, Noh, Kang, Kim, & Lee, 2003). Due to simple molecular structure, anthocyanins are easily absorbed and can reduce the risk of cardiovascular diseases and cancers by exhibiting their anti‐oxidant, anti‐inflammatory, anti‐metastasis, and chemo‐protective properties (Huang, Shih, Chang, Hung, & Wang, 2008; Lazzè et al., 2004). Anthocyaninshave been proven to be capable of scavenging the free radicals and preventing the injuries induced by free radicals (Jiang & Nie, 2014). Multiplebiological activities including the anti‐oxidant (Yang, Yang, & Zheng, 2010), hypolipidemic effect (Liu et al., 2009), and macrophage‐activating effect (Kim et al., 2013), are associated with the phenol content in the mulberry fruit (Donno, Cerutti, Prgomet, Mellano, & Beccaro, 2015; Ercisli & Orhan, 2007; Khan et al., 2013) and non‐anthocyanin polyphenols (Dilip & Tetsuya, 2007; Farrell, Norris, Lee, Chun, & Blesso, 2015; Liang et al., 2012). As a major type of active flavonoid, rutin functions to reduce capillary fragility, improve microcirculation and can be used to treat diabetes mellitus, hypertension, hyperglycemia and other disorders (Duarte, Carvalho, Gadelha, & Braga, 2014; Fernandes et al., 2010).

In recent, different data‐based multivariate approaches have been explored for biological data analysis, which can explore dependency relationships between data sets. These methods include multiple linear regression, principal component regression, partial least squares, and canonical correlation analysis. Among these methods, partial least squares and canonical correlation analysis play a dominating role probably because the extracted latent variable may contribute the biological interpretations of the results. Partial least squares is first developed for process monitoring in chemical industry, exploits the co‐variation between predictor variables and response variables and explores a new set of latent components which are maximally correlated. Canonical correlation analysis is commonly adopted to seek a pair of linear transformations between two sets of variables, and the data are maximally correlated in the transformed space.

At present, the approaches to enhance the quality and improve the nutritional property of mulberry fruit are relatively limited. The detailed composition and the correlation between chemical constituents, minerals and active ingredients of new types of Chinese mulberries should be investigated to explore novel and effective measures and interventions. This study was designed to quantitatively measure the quantity of chemical components, mineral elements and active ingredients, aiming to elucidate the relationship among these ingredients of new types of Chinese mulberries.

## 2 MATERIALS AND METHODS

### 2.1 Material source and processing

Mulberry fruits were harvested from thirteen types of trees at the Xia Jin Planting Basein Shandong in June 2014. The mulberry species included Shenmei098, Baishen, Baiyuwang, Beifanghong, Bingtangshen, Caomeishen, Dabaishen, Wuhedashi, Hanguodabaizhenzhu, Hongguo1, Hongguo2, Ribentianshen and Taiwanguosang cultivars (Table 1). Each cultivar fruit was collected from five trees. All selected trees were picked at the biologically ripe stage, planted for more than 10 years, with high yield and without any pest symptoms. The harvest time was between 20 and 25 May 2015. According to shape and color uniformity, berries were randomly harvested from all cardinally‐oriented branches with different directions around the canopy. The picked fruits were stored at −18°C for subsequent chemical component analysis.

**Table 1:** Variety and source of newly bred and introduced mulberry cultivars

Code	Cultivar	Source
M1	Shenmei098	Linqu County Forestry Bureau of Shandong (new variety)
M2	Baishen	Dezhou Mulberry Cultivation Base located in Xiajin, Shandong (original variety)
M3	Baiyuwang	The Sericultural Research Institute of Shaanxi (new variety)
M4	Beifanghong	The Sericultural Research Institute of Shaanxi (new variety)
M5	Bingtangshen	Shandong Academy of Forestry (new variety)
M6	Caomeishen	Shandong Academy of Forestry (new variety)
M7	Dabaishen	Shandong Academy of Forestry (new variety)
M8	Wuhedashi	Sericultural Research Institute of Guangdong (new variety)
M9	Hanguodabaizhenzhu	Introduced from Korea
M10	Hongguo1	Sericultural Research Institute of Shaanxi (new variety)
M11	Hongguo2	Sericultural Research Institute of Shaanxi (new variety)
M12	Ribentianshen	Introduced from Japan
M13	Taiwanguosang	Introduced from Taiwan

### 2.2 Types of chemical compounds

Chemical compounds included Cyanidin‐3‐glucoside (PubChem CID: 92131208); Vitamin C (PubChem CID: 54670067); Rutin (PubChem CID:5280805);Pectin (PubChem CID: 441476); Copper (PubChem CID: 23978);Iron (PubChem CID: 23925); Calcium (PubChem CID: 5460341); Magnesium (PubChem CID:888); Zinc (PubChem CID: 23994); Potassium (PubChem CID: 813); Selenium (PubChem CID: 6326970).

### 2.3 Chemical component analysis

The concentrations of the following components including moisture, crude fat, crude protein, reducing sugar, anthocyanins, titratable acid, pectin, vitamin C, rutin, Cu, Fe, Ca, Mg, Zn, K, Se, and Na (AOAC, 1990) were determined. Three replicates were performed for each measurement. Sample moisture was determined by drying at 105°C to constant weight. Crude fat was quantitatively assayed according to the AOAC method using soxhlet extraction with absolute ether as a solvent. Crude protein was measured based upon the AOAC method using a Kjeldahl apparatus. Titratable acidity was determined by titrimetric method. A portion of 5 ml of mulberry cultivar juice was diluted in 50 mL of distilled water and titrating to pH 8.2 using 0.1 mol/L NaOH. Anthronecolorimetry was used to quantitatively measure the content of the reducing sugar at a wavelength of 630 nm. A pH‐differential method was adopted to determine the anthocyanin content (the total monomeric anthocyanin content [TMAC]) (Lee, Durst, & Wrolstad, 2005; Souza, Pereira, Queiroz, Borges, & Carneiro, 2012). The absorbance value of the extract was determined at a wavelength of 510 and 700 nm at pH = 1.0 and pH = 4.5. TMAC (expressed as cyanidin‐3‐glucoside) was calculated using the following equations:

(1) $$A={\left(A_{510}-A_{700}\right)}_{\mathrm{pH}=1.0}-{\left(A_{510}-A_{700}\right)}_{\mathrm{pH}=4.5}$$

(2) TMAC=(A×MW×DF×VE×1,000)/(ε×1×M)

where MW is the molecular weight of cyanidin‐3‐glucoside (449 g/mol), DF is the dilution factor, VE is the extraction volume, *ε* is the molar extinction coefficient of cyanidin‐3‐glucoside (29,600), and *M* is the quantity of extracted berry.

Pectin was determined using pyridine colorimetry (Pang et al., 2012). Ultraviolet chromatometry was used to determine the content of rutin. The 2,4‐Dinitrobenzene hydrazine colorimetry was used to analyze the content of vitamin C. For the mineral‐substance analysis, 1 g sample was added to a digestion tank, supplemented with perchloric acid and nitric acid at a ratio of 1:4.The digestion tank was placed in a dry box at 100°C for 1 hr, and then at 130°C for 2 hr, followed by cooling. The sample solution was transferred to a 50 ml volumetric flask and diluted with high‐purity water, supplemented with Ca with 10% strontium chloride. A standard curve was generated based on the standard working solution using Cu, Fe, Ca, Mg, Zn, K, Se, and Na. The concentrations of anthrone, glucose, sulfuric acid, sodium hydroxide, hydrochloric acid, cupric sulfate, potassium sulfate, ammonium sulfate, methyl blue, methyl red, hydrogen peroxide, selenium, orthoboric acid, vitamin, C,2,4‐dinitrophenylhydrazine, methanol, ethanol, sodium nitrite, aluminum nitrate, activated carbon, citric acid, potassium biphthalate, rutin reference substance, Cu, Fe, Ca, Mg, Zn, K, and Se were quantitatively analyzed by Sinopharm Chemical Reagent, Shanghai, China.

### 2.4 Statistical analysis

The average values were obtained from three parallel experiments for each type of mulberry fruit. The results are expressed as means ± standard deviation. Data were subject to standardization. The partial‐least‐squares (PLS) and principal component analysis (PCA) were employed using the Unscrambler software package (Version 9.7; CAMO, Trondheim, Norway).The PLS was used to detect cause–effect relationship, and the correlation coefficient (*R* ^2^) and root‐mean‐square error of cross validation (RMSECV) were used to establish a model to evaluate the effect of total phenols, anthocyanins and other related components. Canonical‐correlation analysis was used to analyze the correlation between the mineral elements and chemical components (DPS7.0 software).

## 3 RESULTS

### 3.1 Analysis of main chemical components and mineral elements

As illustrated in Table 2, the contents of moisture, crude fat, crude protein, reducing sugar, anthocyanin, titratable acid, pectin, vitamin C and rutins of the mulberries were approximately (76.75–90.55)%, (0.35–1.97)%, (0.51–1.77)%, (0.24–0.68)%, (0–0.34)%, (1.10–5.84) mg/g, (2.88–6.13) mg/g, (0.87–4.08) mg/g, and (0–0.32) mg/g, respectively. The contents of anthocyanin and rutins were consistent with the fruit color. A low quantity of anthocyanin and rutins were detected in the white or light color fruits. The contents of Cu, Fe, Ca, Mg, Zn, K, and Se were (0–0.50), (7.72–30.13), (180.61–423.30), (13.96–34.04), (4.06–10.58), (87.70–208.44), and (1.80–5.82) μg/g, respectively, as demonstrated in Table 3.

**Table 2:** Comparison of chemical composition among different cultivars of mulberry fruits

Code	Cultivar	Moisture (g/100 g)	Crude fat (g/100 g)	Crude protein (g/100 g)	Reducing sugar (g/100 g)	Anthocyanin (g/100 g)
M1	Shenmei098	85.64 ± 1.43	0.56 ± 0.04	0.96 ± 0.12	0.35 ± 0.01	0.15 ± 0.06
M2	Baishen	82.85 ± 0.73	1.41 ± 0.08	0.66 ± 0.16	0.24 ± 0.01	0.00 ± 0.00
M3	Baiyuwang	83.58 ± 0.74	0.99 ± 0.04	0.99 ± 0.17	0.38 ± 0.01	0.00 ± 0.00
M4	Beifanghong	82.33 ± 0.35	1.44 ± 0.06	0.96 ± 0.18	0.29 ± 0	0.21 ± 0.01
M5	Bingtangshen	77.46 ± 0.55	0.96 ± 0.09	1.77 ± 0.10	0.55 ± 0.05	0.13 ± 0.00
M6	Caomeishen	78.75 ± 0.4	1.31 ± 0.04	1.02 ± 0.07	0.68 ± 0.01	0.01 ± 0.01
M7	Dabaishen	76.75 ± 1.76	1.11 ± 0.02	1.00 ± 0.07	0.37 ± 0.02	0.00 ± 0.00
M8	Wuhedashi	79.46 ± 0.41	0.35 ± 0.02	1.24 ± 0.39	0.54 ± 0.02	0.27 ± 0.00
M9	Hanguodabaizhenzhu	81.00 ± 0.43	1.50 ± 0.14	1.19 ± 0.14	0.51 ± 0.02	0.01 ± 0.00
M10	Hongguo1	90.55 ± 0.55	0.34 ± 0.02	0.51 ± 0.15	0.25 ± 0.01	0.27 ± 0.00
M11	Hongguo2	86.11 ± 2.09	1.29 ± 0.13	0.59 ± 0.07	0.28 ± 0.01	0.34 ± 0.01
M12	Ribentianshen	80.71 ± 0.7	0.76 ± 0.13	0.99 ± 0.13	0.4 ± 0.01	0.31 ± 0.00
M13	Taiwanguosang	82.13 ± 1.52	1.97 ± 0.04	0.81 ± 0.11	0.33 ± 0.01	0.21 ± 0.01

**Table 3:** Comparison of mineral elements among different varieties of mulberry fruits (μg/g)

Code	Cultivar	Cu	Fe	Ca	Mg	Zn	K	Se
M1	Shenmei098	0.04 ± 0.06	15.10 ± 0.08	226.15 ± 16.98	21.00 ± 0.01	4.49 ± 0.16	132.61 ± 1.09	2.17 ± 0.13
M2	Baishen	0.09 ± 0.07	9.62 ± 0.20	303.61 ± 16.54	25.32 ± 0.05	5.62 ± 0.15	154.40 ± 0.97	3.14 ± 0.16
M3	Baiyuwang	0.50 ± 0.00	30.13 ± 0.57	276.50 ± 6.69	24.81 ± 0.06	9.37 ± 0.02	155.96 ± 0.24	1.80 ± 0.10
M4	Beifanghong	0.13 ± 0.00	7.72 ± 0.10	343.33 ± 28.97	26.29 ± 0.03	5.77 ± 0.13	166.06 ± 1.58	3.58 ± 0.38
M5	Bingtangshen	0.40 ± 0.10	15.00 ± 0.39	390.15 ± 35.95	33.38 ± 0.00	9.49 ± 0.11	203.25 ± 0.08	3.57 ± 0.20
M6	Caomeishen	0.05 ± 0.09	10.35 ± 0.25	395.19 ± 47.17	30.64 ± 0.11	7.64 ± 0.23	191.89 ± 1.59	4.44 ± 0.05
M7	Dabaishen	0.35 ± 0.00	16.02 ± 0.36	380.24 ± 27.70	34.04 ± 0.06	10.58 ± 0.32	208.44 ± 0.84	4.10 ± 0.13
M8	Wuhedashi	0.00 ± 0.00	13.87 ± 0.21	386.65 ± 47.28	27.28 ± 3.42	6.69 ± 0.10	183.07 ± 0.46	5.82 ± 0.07
M9	Hanguodabaizhenzhu	0.00 ± 0.00	15.00 ± 0.11	423.30 ± 25.83	28.24 ± 0.06	7.97 ± 0.07	170.58 ± 0.66	4.01 ± 0.11
M10	Hongguo1	0.07 ± 0.00	9.30 ± 0.05	180.61 ± 3.50	13.96 ± 0.03	4.06 ± 0.37	87.70 ± 0.60	2.48 ± 0.03
M11	Hongguo2	0.32 ± 0.00	14.24 ± 0.08	226.32 ± 31.15	20.46 ± 0.05	5.32 ± 0.07	131.58 ± 0.79	1.54 ± 0.10
M12	Ribentianshen	0.00 ± 0.00	9.14 ± 0.34	390.59 ± 7.91	28.31 ± 0.03	5.50 ± 0.06	170.40 ± 0.49	5.78 ± 0.28
M13	Taiwanguosang	0.45 ± 0.08	14.41 ± 0.18	418.70 ± 35.18	26.39 ± 0.02	9.12 ± 0.15	165.36 ± 1.57	5.29 ± 0.03

### 3.2 Regression analysis of anthocyanins and rutins with other chemical components

**Figure 1:**
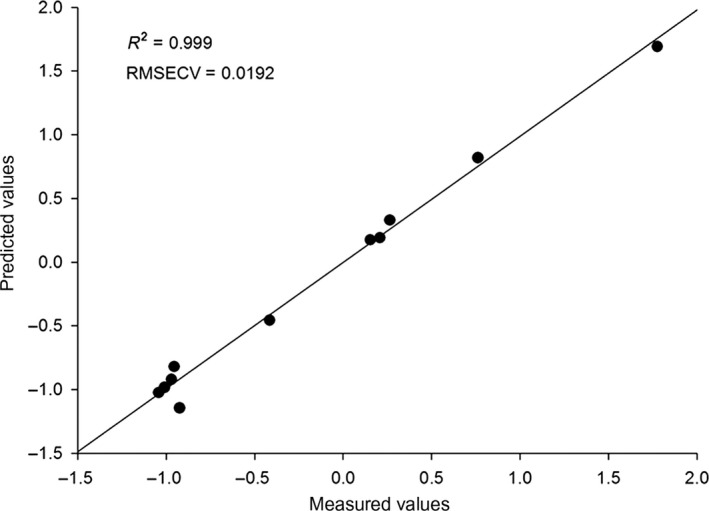
Linear regression plot of measured versus predicted content of rutins of mulberry samples

**Figure 2:**
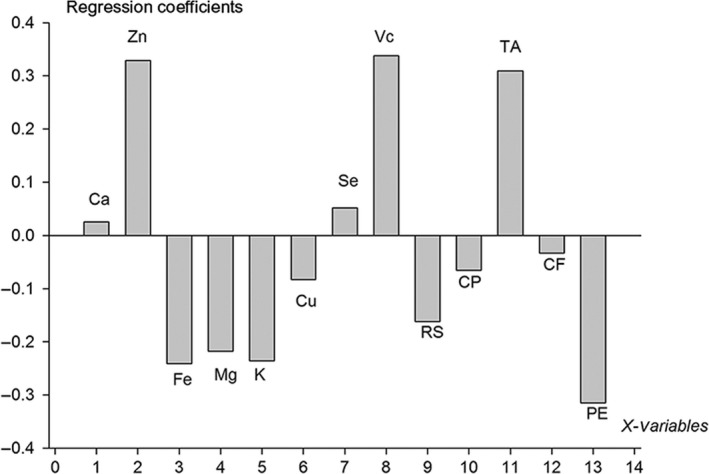
Regression coefficients of rutins versus Ca, Zn, Fe, Mg, K, Cu, Se, vitamin C (Vc), reducing sugars (RS), crude protein (CP), titratable acid (TA), crude fat (CF), and pectin (PE)

The relationship between each chemical component and the rutin content was illustrated in Figure 1. Principal divisor 1 of the PLS accounted for 75.69% in the combined data. The RMSECV was low (0.0192), and the correlation index reached 0.999. Zn, vitamin C, and titratable acid were positively correlated with rutins (Figure 2). The content of Ca was weakly positively correlated with Se.

**Figure 3:**
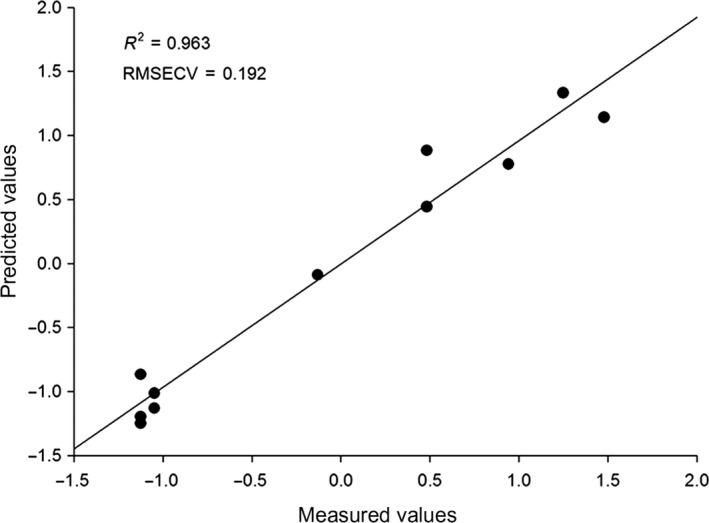
Linear regression plot of measured versus predicted content of anthocyanin of mulberry samples

**Figure 4:**
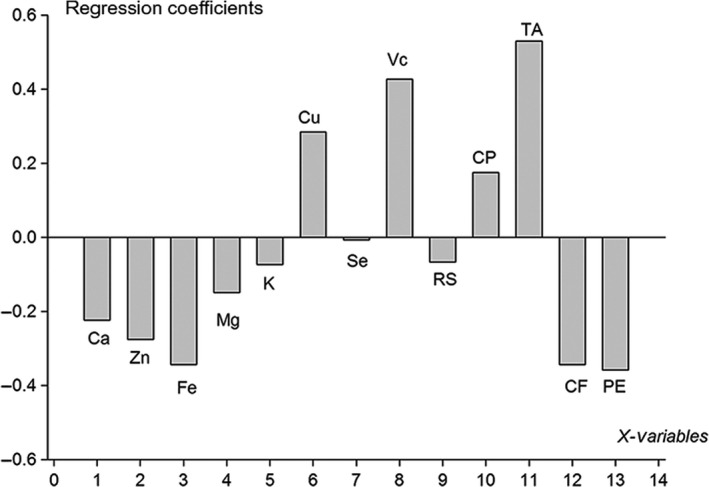
Regression coefficients of anthocyanin versus Ca, Zn, Fe, Mg, K, Cu, Se, vitamin C (Vc), reducing sugars (RS), crude protein (CP), titratable acid (TA), crude fat (CF), and pectin (PE)

The quantity of Fe, Mg, K, reducing sugar and pectin was negatively correlated with rutins. The quantity of Cu, crude proteins was weakly negatively associated with crude fat. The relationship between chemical components and anthocyanin was illustrated in Figure 3. Principal divisor 1 of the PLS accounted for 63.73% in the combined data. The RMSECV was 0.192 and the correlation index reached 0.963. Cu, vitamin C, crude protein, and titratable acid were positively correlated with anthocyanin, whereas Ca, Zn, Fe, Mg, crude fat, and pectin were negatively correlated with anthocyanin. The quantity of K and Se was weakly negatively correlated with reducing sugar (Figure 4).

### 3.3 PCA of chemical components

Principal components of eight indices were evaluated (Table 4). Kaiser–Meyer–Olkin value was 0.691 and the level of significance was 0.093, indicating that these data could be analyzed by PCA. Based on the value‐greater‐than‐1.0 rule, three principal components were identified by the varimax rotation method, which explained 80.60% of the general data of the sample. The first principal components included anthocyanin, vitamin C, titratable acid and rutins. According to the loading coefficient between the indices and the first principal components, this component was designated the “activity factor,” which accounted for 36.24% of the general data of the sample. The second principal components consisted of reducing sugar and crude protein, which explained 23.39% of the general data of the sample. The third principal components included crude fat and pectin, which accounted for 20.96% of the general data of the sample.

**Table 4:** PCA of chemical components

Chemical components	*f*1	*f*2	*f*3
Reducing sugar	−0.242	0.876	−0.159
Crude protein	−0.171	0.904	−0.067
Vitamin C	0.813	−0.239	0.172
Anthocyanin	0.861	−0.120	−0.207
Titratable acid	0.838	−0.103	−0.036
Crude fat	−0.170	0.021	0.913
Pectin	−0.017	−0.409	0.773
Rutin	0.824	−0.191	−0.379

### 3.4 PCA of mineral elements

Principal components of seven indices were analyzed in Table 5. Kaiser–Meyer–Olkin value was 0.662 and the level of significance was 0.000, suggesting that these data could be analyzed by PCA. Based on the eigenvalue‐greater‐than‐1.0 rule, two principal components were identified by the varimax rotation method, which accounted for 86.871% of the general data of the sample. The first principal components included Ca, Mg, K and Se, which represented 52.44% of the general data of the sample. The second components consisted of Cu, Fe and Zn, which represented 34.25% of the general data of the sample.

**Table 5:** Mineral element PCA

Mineral element	*f*4	*f*5
Cu	0.030	0.893
Fe	−0.088	0.872
Ca	0.968	−0.039
Mg	0.933	0.219
Zn	0.581	0.759
K	0.938	0.218
Se	0.798	−0.423

### 3.5 Canonical‐correlation analysis of chemical components and mineral elements

From the PCA, three chemical principal components including the activity factor (*f*1),the reducing sugar and crude protein (*f*2) and the crude fat and pectin (*f*3) were identified. Two mineral principal components of Ca plus three other mineral elements (*f*4) and Cu plus two other mineral elements (*f*5) were obtained. The canonical‐correlation analysis between chemical components and mineral elements of these five principal components was performed (Table 6).

**Table 6:** Principal‐component correlation coefficient

Principal component	*f*1	*f*2	*f*3
*f*4	−0.1648	0.6957	0.4200
*f*5	−0.4451	0.0761	0.0870

### 3.6 Correlation coefficient matrix

By maximizing variance varimax rotation in the PCA, the correlation index of each principal ingredient from the same group was zero, suggesting that no principal ingredients could be substituted. The principal *f*1 of chemical components was negatively correlated with that of mineral elements, indicating that the mineral elements exerted a weak inhibitory effect on vitaminC, anthocyanin, rutins and titratable acid. The *f*2/*f*3 was positively correlated with *f*4, suggesting that reducing sugar, crude protein, crude fat, and pectin were significantly correlated with mineral elements (Table 6).

### 3.7 Canonical‐correlation‐coefficient analysis

The first canonical variable was qualified the significance test (*α* = 0.01) with a canonical‐correlation coefficient of 0.859, whereas the second canonical variable failed to achieve the significance test (*α* = 0.01) with a canonical correlation of 0.401. A canonical‐correlation coefficient existed between chemical components and mineral elements, which was validated by analyzing the first canonical variables (Table 7).

**Table 7:** Canonical‐correlation‐coefficient analysis

Number	Canonical correlations	Wilk's	Chi‐SQ	*df*	*p*
1	0.8590	0.2198	13.6341	6	0.0340
2	0.4013	0.8389	1.5807	2	0.4537

### 3.8 Canonical‐correlation‐structure analysis

**Figure 5:**
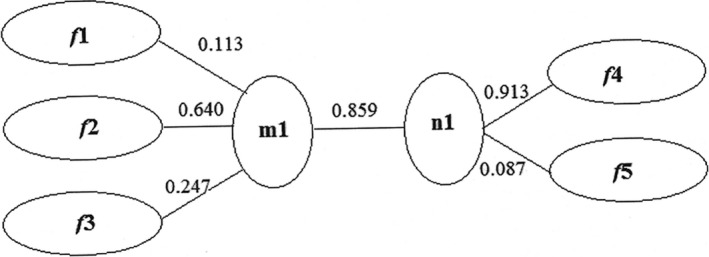
Canonical‐correlation analysis of chemical components and mineral elements

To analyze the relative effects of principal components between two groups when they formed a canonical variable, it was necessary to observe the first canonical variable: *m*1 = 0.113*f1 *+* *0.640*f*2 + 0.247*f*3. For the first canonical variety of chemical components, *f*2 (0.640) was most evident among the correlated indexes of each principal ingredient, suggesting that reducing sugar and crude fat affected the quality of mulberries. The second evident index was *f*3 (0.247), which was the smallest at 0.113. For the first canonical variety of mineral elements, *n*1 = 0.913*f*4* *+* *0.087*f*5. For the first canonical variety of mineral elements, Ca, Mg, K, and Se exerted the most significant effect among the correlated indexes of two principal ingredients, which could be considered as the principal effective mineral elements of mulberries. As illustrated in Figure 5, unbalanced load coefficients were observed among *f*1, *f*2, and *f*3 in the first canonical variables of chemical components, indicating that reducing sugar and crude proteins were correlated with the first canonical variables of chemical components. The load coefficients *f*4 and *f*5, especially *f*4, significantly differed in the first canonical variables of mineral elements. Because of the importance of the first canonical variable (0.859), reducing sugar and crude proteins exerted significant effect upon the contents of Ca and other three mineral elements, suggesting significant correlation between mineral elements (Ca, Mg, K, and Se) and chemical components (reducing sugar and crude proteins).

## 4 DISCUSSION

In this investigation, the new type of Chinese mulberry fruits possesses high nutritional value, whereas the chemical components and mineral ingredients significantly differ among types. The content of rutins in dark Hongguo1 mulberries reached up to 0.32 mg/g, which is higher than the value previously reported (Ercisli & Orhan, 2007). The quantity of anthocyanin contained in Hongguo 2 mulberries was 0.34 g/100 g, whereas it was undetectable in Baishen mulberries (light mulberries). The quantity of the rutins and anthocyanin contained in these mulberries presents with identical changing pattern, which is consistent with previous findings (Donno et al., 2015; Ercisli & Orhan, 2007).

Appropriate understanding of the chemical composition of mulberry ruits can explore and identify novel resources of natural antioxidants. (Kara & Erçelebi, 2013; Wang, Xiang, Wang, Tang, & He, 2013). PCA is primarily used as a tool in exploratory data analysis and for establishing predictive models, and for visualizing genetic distance and relatedness between populations. PCA can be performed by eigenvalue decomposition of a data covariance matrix or singular value decomposition of a data matrix. In this study, PCA demonstrated that the content of rutins, anthocyanin, and vitamin C was positively correlated with titratable acid. The level of titratable is one of the most important factors that determine the flavor of mulberry fruits, and plays a key role in the stability among anthocyanin, vitamin C and rutins. Anthocyanin is inclined to degrade when the degree of acidity declines. Fruit color is a critical index related to the quality of mulberry fruits. Specifically, the mulberry fruits with a dark color are high in the contents of anthocyanin and rutin, indicating high quality of mulberry fruits.

The resistance of mulberries to oxidative stress is positively correlated with the content of totalphenolic compounds, anthocyanin and rutin. Consequently, how to improve quantity of the active elements, particularly those related to the effect of mineral elements on fruit quality has been extensively investigated. Previous studies (Garcia‐Banuelos, Hermosillo‐Cereceres, & Sanchez, 2011) have evaluated the specific varieties and growing conditions, such as the changes in the mineral elements that are caused by soil or irradiance. Nevertheless, the relationship among these active elements has been rarely studies.

In this investigation, we identified seven mineral elements which were related to human health and analyzed the relationship among these mineral elements and alternative chemical components. The correlation analysis demonstrated that Cawasone of the most pivotal mineral elements, followed by K, Mg, Fe, Zn, Se, and Cu, respectively. Among them, the quantity of Se was positively correlated with Ca, Mg and K, whereas the content of Zn was positively associated with Cu and Fe. Se is an important trace element that possesses anti‐cancer effect because it plays a role in suppressing the growth and proliferation of cancer cells, which are produced by the disintegration of polyunsaturated fat in the human body. Additionally, Se and vitamin C confer mulberries with health benefits owing to their synergistic effects upon oxidative resistance (Garcia‐Banuelos et al., 2011).Therefore, mulberries deserve to be further investigated from the perspective of human nutritional requirement. Due to abundant content of anthocyanin, rutins, Ca, Se, Fe, and pectin, mulberries can be consumed as a valuable source of rich nutritional components.

Canonical‐correlation analysis demonstrated that multiple ingredients, such as reducing sugar, crude protein, crude fat, and pectin are intimately correlated with mineral elements, and play a coordinated role. However, relatively weak correlation is observed between mineral elements and the accumulation of active ingredients. Se and Cu exert a significant effect upon the function of active ingredients.

Besides the genetic and physiological influence, chemical components and nutrition of the mulberry fruits are probably affected by the environmental factors, such as the soil chemical properties and climatic conditions, agronomic conditions including the harvesting techniques during different stages of maturity and technical factors, such as disposal after harvesting and conditions for processing and storage (Donno et al., 2012, 2012; Sadia et al., 2014). Taken together, our findings add evidence to the modification of farming methods, aiming to improve the quality and nutritional components of mulberry fruits. Moreover, the findings obtained from this investigation offers reference for the selection of the mulberry variety.

## CONFLICT OF INTEREST

None declared.
